# Validation of the Brazilian Oral Health Literacy-Adults Questionnaire

**DOI:** 10.3928/24748307-20220822-01

**Published:** 2022-07

**Authors:** Eliete Rodrigues Almeida, Mohammad Mehdi Naghibi Sistani, Cristiane Baccin Bendo, Isabela de Almeida Pordeus, Ramon Targino Firmino, Saul Martins Paiva, Fernanda Morais Ferreira

## Abstract

**Background:**

Recognizing that a deficit of reading and numeracy skills is associated with poorer oral health, contemporary researchers have identified additional components as important attributes of oral health literacy (OHL). So, the use of comprehensive functional OHL tools is crucial. The Oral Health Literacy-Adults Questionnaire (OHL-AQ) evaluates reading comprehension, numeracy, listening and decision-making skills.

**Objective:**

Describe the validation process of the OHL-AQ Brazilian version (BOHL-AQ).

**Methods:**

The cross-culturally adapted version, BOHL-AQ, was applied to 180 employees age 18 to 71 years (mean = 37.2; standard deviation [*SD*] = 11.7) from a private university located in the Southeast of Brazil. Psychometric properties were evaluated through the analysis of internal consistency (Cronbach's alpha), reproducibility (test-retest), convergent validity (BREALD-30; education level), discriminant validity (family income; dental services), predictive validity (self-perception; literacy questions) and construct validity (Exploratory Factor Analysis). Confirmatory Factor Analysis (CFA) evaluated the dimensionality of the BOHL-AQ, with Promax method for rotation. Data were analyzed using SPSS Statistics software and the Mplus program.

**Key Results:**

BOHL-AQ mean score = 11.84 (*SD *= 3.1); administration mean time = 8 minutes (*SD* = 1.6); good internal reliability (Cronbach's alpha = 0.73) and excellent reproducibility (kappa = 0.89; intraclass correlation coefficient = 0.97). Preliminary tests showed that data were suitable for PCA (Kayser-Meyer-Olkin measure = 0.75; Barlett's Test of Sphericity significant [*p* < .001]). CFA showed that the instrument had a four-factor solution with excellent model fit estimates (χ^2^ = 636.587154.16, *p* value = .00117, Comparative Fit Index = 0.9787, Tucker Lewis index = 0.97, and Root Mean Square Error of the Approximation = 0.03). BOHL-AQ high scores significantly correlated to high education level, dental visit within the last year and for preventive reason, more independence and self-confidence on reading and filling out health forms, and better oral health self-perception (*p* < .05).

**Conclusions:**

The BOHL-AQ showed to be a fast and reliable instrument to assess a comprehensive functional OHL at Brazilian community and clinical settings. [***HLRP: Health Literacy Research and Practice*. 2022;6(3):e224–e231.**]

**Plain Language Summary:**

Recognizing the need of advancing knowledge related to OHL, this study aimed to describe the validation process of the BOHL-AQ. Cross-cultural adaptation and psychometric properties evaluation presented satisfactory results. The BOHL-AQ proved to be a fast and valid instrument to measure comprehensive functional OHL in the Brazilian context.

Literacy skills are required for health information acquisition, appraising, and applying its concepts in order to make health decisions. This process is recognized as health literacy (HL) and can be divided in three types: functional health literacy (reading and writing skills), interactive health literacy (skills that allow individuals to extract information and apply to changing life circumstances), and critical health literacy (cognitive and social skills needed for critically analyzing and using health information to personal situations) (van der Heide et al., 2015). Most studies have focused on functional HL. However, increasing attention has been paid on interactive and critical HL, because the different types of health literacy vary in their relevance for patients' ability to exert control in own care (Sykes et al., 2013; van Heide et al., 2015). Healthy People 2030 recently included HL as a social determinant of health, and expanded the individual concept, high-lighting that physicians, as both clinicians and organizational leaders, have the key roles to play in helping individuals and organizations become health literate, attaining health literacy for all (Brach & Harris, 2021). Moreover, Stacey et al. (2017) highlighted the importance of interventions that support patients´ decision-making, providing adequate information to clarify personal decisions, called “decision aids.” People exposed to decision aids seems to have more accurate risk perceptions (Stacey et al., 2017). Thus, research is needed to investigate the role of health literacy and its measurement in strategies based on decision aids, improving professional-patient relationship. The concept of oral health literacy (OHL) is similar to health literacy but is specific to oral health. OHL has been gained the attention of policy makers due to its proven effect on oral health outcomes (Praveen et al., 2021). Individuals with low OHL have presented higher risk of oral diseases (Batista et al., 2017; Guo et al., 2014), showing the importance of OHL levels identification in the development of public health programs, by creating appropriate educational materials and actions. Several OHL instruments have been developed, but most of them have limitations (Dickson-Swift et al., 2014; Parthasarathy et al., 2014). To create a more general instrument, Sistani et al. (2014) proposed the Oral Health Literacy-Adult Questionnaire (OHL-AQ), evaluating numeracy, reading comprehension and two additional skills: listening and decision-making. The OHL-AQ was designed in Iran, translated to the English language (Sistani et al., 2014) and subsequently validated to the English-speaking population (Flynn et al., 2016). There are actually five OHL Brazilian instruments: Brazilian Rapid Estimate of Adult Literacy in Dentistry-30 (BREALD-30) (Junkes et al., 2015), Brazilian Rapid Estimate of Adult Literacy in Medicine and Dentistry-20 (Cruvinel et al., 2017), both evaluating word recognition; the Brazilian version of the Oral Health Literacy Assessment (BOHL-AQ) in Spanish (Bado et al., 2018), which assess reading, pronunciation and comprehension; Brazilian version of the Health Literacy in Dentistry-14 (Mialhe et al., 2020), evaluating comprehension of oral health information; and the Brazilian Hong Kong Oral Health Literacy Assessment Task for Paediatric Dentistry (BOHLAT-P) (Firmino et al., 2020), that measures oral health knowledge, numeracy and reading comprehension of parents. However, the authors considered the BOHLAT-P very long and suggested a short version. Despite the importance of these tools, there is still a lack of practical instruments. The OHL-AQ (Sistani et al., 2014) seems to be a fast and comprehensive functional OHL instrument. Therefore, the aim of this study was to validate the Brazilian version of the OHL-AQ (BOHL-AQ)

## Methods

### Ethics

The study was approved by the Human Research Ethics Committee from the Cruzeiro do Sul University (protocol #68527417.4.0000.8084), which was conducted in compliance with the ethical principles of the World Medical Association Declaration of Helsinki.

### Instrument

The OHL-AQ is a questionnaire comprising 17 items in four sections: the reading comprehension consists of three incomplete sentences on oral health knowledge. Five possible choices were offered for completing each sentence, and the respondents were required to fill in the blanks. Numeracy consisted of four questions related to two topics, amoxicillin consumption prescription and instructions for sodium fluoride mouth rinse. The prescription and instructions were added in a written box, and participants were instructed to write or select the answers. Listening was assessed by the interviewer reading post-extraction instructions, followed by one free-text response and one multiple choice question. The decision-making section contained five questions related to common oral health problems. Participants were required to read the questions and select one of the four possible choices for each question. The interviewer should not help participants in reading, answering, or in the conceptual meaning of items, but they could explain how to complete the questionnaire. They should also check for missing items and ask the participants to answer those or to select the *do not know* alternative. Correct answers were scored 1, and incorrect answers 0, with a summed OHL-AQ score ranging from 0 to 17 points. The OHL-AQ scores were categorized into three groups: inadequate 0 to 9; marginal 10 to 11; and adequate 12 to 17 (Sistani et al., 2014).

### Cross-Cultural Adaptation

This process was systematically performed after authorization of the original OHL author (Sistani et al., 2014), following standard recommendations described on previous Brazilian OHL studies (Bado et al., 2018; Cruvinel et al., 2017; Firmino et al., 2020; Junkes et al., 2015; Mialhe et al., 2019) and the COSMIN (COnsensus-based Standards for the selection of health Measurement INstruments) checklist (Mokkink et al., 2010). The first stage consisted of the translations to the Brazilian language, by two Brazilian bilingual translators (one dentist, with knowledge on the instrument terms, and one English professor, without dentistry knowledge). For the conceptual equivalence, an expert panel, composed by four university dentistry professors with experience on oral health education evaluated the two questionnaires regarding the concepts that would be relevant and pertinent to the Brazilian cultural context, the meaning of words in both languages and the effects in different cultures. We searched for possible discrepancies and developed a synthetized translated version. An independent translator (born and literate in an English-speaking country, with linguistic and cultural mastering of both English and Brazilian Portuguese) back-translated this synthetized version to the English language. The translator was not aware of the research's objectives and did not have access to the OHL-AQ, that was subsequently sent to the original instrument author (Sistani et al., 2014), who did not modify any item. So, the equivalence between items from the back-translated version and the original instrument evidenced that instruments remained equivalent. Semantic equivalence was also evaluated by the experts, through the analysis of the referential and general meaning. Two aspects were considered to determine words replacement: ambiguity and readability (Junkes et al., 2015). Conceptual equivalence of the items sought to demonstrate the relevance and acceptability of the instrument, and it was obtained by the pre-test, comprising 20 employees that did not participated to the study sample, by face-to-face interviews. The pre-test aimed to check participants understanding in terms of clarity, completion time and possible issues. There were no difficulties on understanding the items during the interviews. The cross-cultural adaptation process enabled the BOHL-AQ after authorization of the expert panel.

### Psychometric Properties Evaluation

We applied the BOHL-AQ to 180 employees age 18 to 71 years from a private university located in the Southeast of Brazil (Sao Paulo city). Exclusion criteria included individuals that were unable to read or write in Brazilian Portuguese language, illiterate and those with cognitive impairment, uncorrected vision, hearing impairment or obvious signs of intoxication by drugs or alcohol at the time of interview. The sample size was calculated using an online calculator, with the following parameters: type I error probability = 0.05, power = 0.80, number of items = 17, expected value for Cronbach's alpha coefficient = 0.72 (Sistani et al., 2014), required value for Cronbach's alpha coefficient = 0.80, resulting in a minimum sample of 150 individuals. Adding up 20% of missing data/individuals, the final sample comprised 180 individuals, in compliance with the recommendation to include from 5 to 10 respondents for each question under validation (Anthoine et al., 2014). The University Department of Human Resources provided a list of employees, including faculties, administrative and cleaning staff. Sample was drawn from this list and stratified according to the education level, following the Brazilian Institute of Geography and Statistics classification, regarding data from Sao Paulo city (Instituto Brasileiro de Geografia e Estatística, 2018): incomplete elementary school (38.1%); incomplete high school (18.6%); incomplete undergraduate (27%); complete undergraduate and postgraduate (16.3%). A single investigator (E.R.A.), previously trained, conducted the interviews, applying the BOHL-AQ, the BREALD-30 (Junkes et al., 2015), and a questionnaire addressing (covariates): marital status (married/otherwise), race and ethnicity (White people and Black, Indigenous, People of Color), sex (male/female), education level (until 8 years of study [elementary school]/more than 8 years of study), last dental visit (in the last year/more than 1 year ago) and its reason (treatment/pain-emergency/prevention), self-perception on oral health (*good* [excellent, very good, good or fair] or *poor*), age (≤29 years; 30–44 years; ≥45 years old), monthly household income (<$296.55; $296.55–553.56; >$553.56), and questions related to literacy skills (“How often do you need help to read clinical/hospital materials?” [*always*, *sometimes*, *rarely*, *never*] and “How confident do you feel when you are filling out forms?” [*not confident*, *a little confident*, *extremely confident*]).

### Statistical Analysis

Reliability was assessed in two ways: internal consistency and reproducibility. Internal consistency was tested using Cronbach's alpha. Values ≥0.70 were considered acceptable (Cronbach, 1951). Reproducibility was assessed by test-retest based on an item-by-item (Kappa coefficient) and intraclass correlation coefficient (ICC). The instrument was reapplied in 10% of the sample 1-month later. The ICC was categorized (Kline, 2011): ≤0.40 weak correlation; 0.41–0.60 moderate; 0.61–0.80 good; 0.81–1.00 excellent correlation. Convergent validity was accessed by comparing OHL-AQ to BREALD-30 scores and education level (χ^2^ test). Discriminant validity was tested by comparing OHL-AQ according to groups of age, sex, ethnicity, income, and dental services (reason and time of the last dental visit) (χ^2^ test). For predictive validity, oral health self-perception and literacy questions were used (χ^2^ test). Exploratory factorial analysis (EFA) was employed to assess the construct validity. The suitability of data set was verified through the Kayser-Meyer-Olkin measure (KMO) (>0.50) and Barlett's Test of Sphericity (*p* < .05). Principal Components Analysis (PCA) was used to extract factors based on Kayser's criteria (Eingenvalues >1) as well as a visual inspection of the scree plot. Promax method was used for rotation. Factor loadings equal to or greater than 0.40 were considered adequate (Nunnaly & Bernstein, 1994). To confirm the dimensionality of the OHL-AQ suggested in EFA, as well as to evaluate model fit, a confirmatory factor analysis (CFA) was performed. The goodness of fitting of the model was evaluated by the following statistical parameters: Chi-square (χ^2^) statistic, Comparative Fit Index (CFI), Tucker Lewis index (TLI) and Root Mean Square Error of the Approximation (RMSEA). CFI and TLI values ≥0.95, and RMSEA values ≤0.06 indicate an excellent model fit (Kline, 2011). Data analysis was performed using SPSS Statistics version 25.0 and the Mplus program (version 8.2) for the CFA. The significance level of all analysis was set at 5%.

## Results

Validity and reliability assessment were conducted in a sample of 180 adults age 18 to 71 years (mean age = 37.2; *SD* = 11.7), 71.6% female and 55.6% with incomplete high school education. BOHL-AQ mean score = 11.84 (*SD* = 3.1), administration mean time = 8 minutes (*SD* = 1.6). The instrument demonstrated good reliability. The internal consistency of the total scale was adequate (Cronbach's alpha = 0.735), with an excellent reproducibility (kappa = 0.89) (**Table 1**).

**Table 1:**
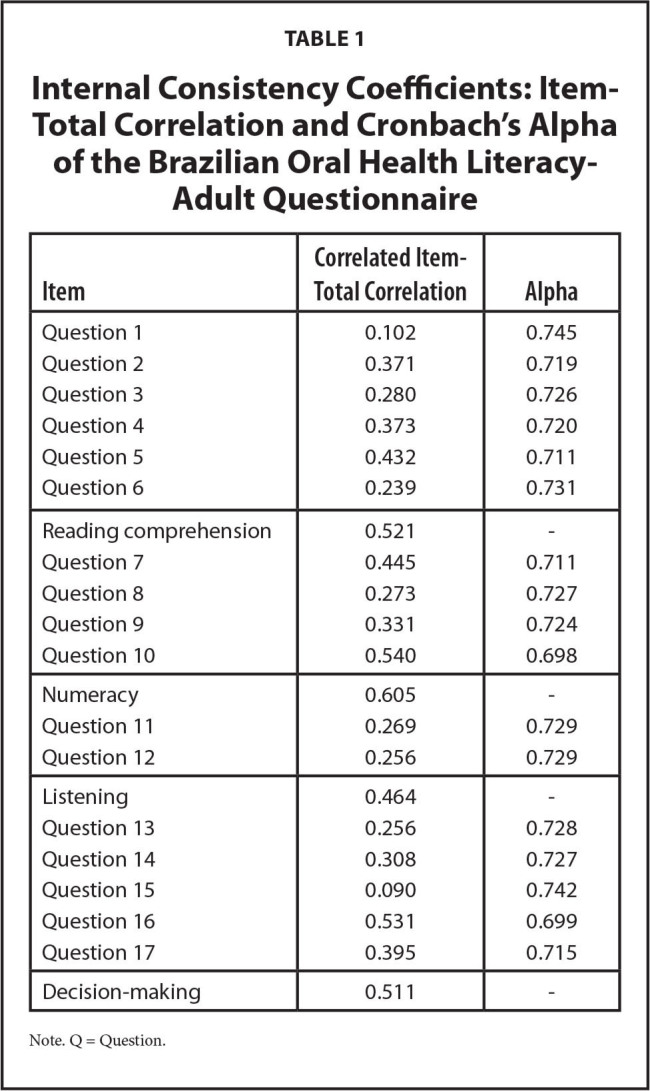
Internal Consistency Coefficients: Item-Total Correlation and Cronbach's Alpha of the Brazilian Oral Health Literacy-Adult Questionnaire

**Item**	**Correlated Item-Total Correlation**	**Alpha**

Question 1	0.102	0.745
Question 2	0.371	0.719
Question 3	0.280	0.726
Question 4	0.373	0.720
Question 5	0.432	0.711
Question 6	0.239	0.731

Reading comprehension	0.521	-
Question 7	0.445	0.711
Question 8	0.273	0.727
Question 9	0.331	0.724
Question 10	0.540	0.698

Numeracy	0.605	-
Question 11	0.269	0.729
Question 12	0.256	0.729

Listening	0.464	-
Question 13	0.256	0.728
Question 14	0.308	0.727
Question 15	0.090	0.742
Question 16	0.531	0.699
Question 17	0.395	0.715

Decision-making	0.511	-

Note. Q = Question.

The BOHL-AQ demonstrated satisfactory convergent validity. BOHL-AQ levels were significantly correlated to the BREALD-30 scores and education level (*p* < .001). The proportion of adults classified as OHL inadequate, marginal, and adequate differed on comparing to age, race and ethnicity, monthly household income, reason and time of the last dental visit (discriminant validity; *p* < .05). Individuals who self-declared more independent and confident on reading and filling out health forms, as well as those with better self-perception on oral health, were more likely to pertain to the adequate OHL level (predictive validity; *p* < .05) (**Table 2**).

**Table 2:**
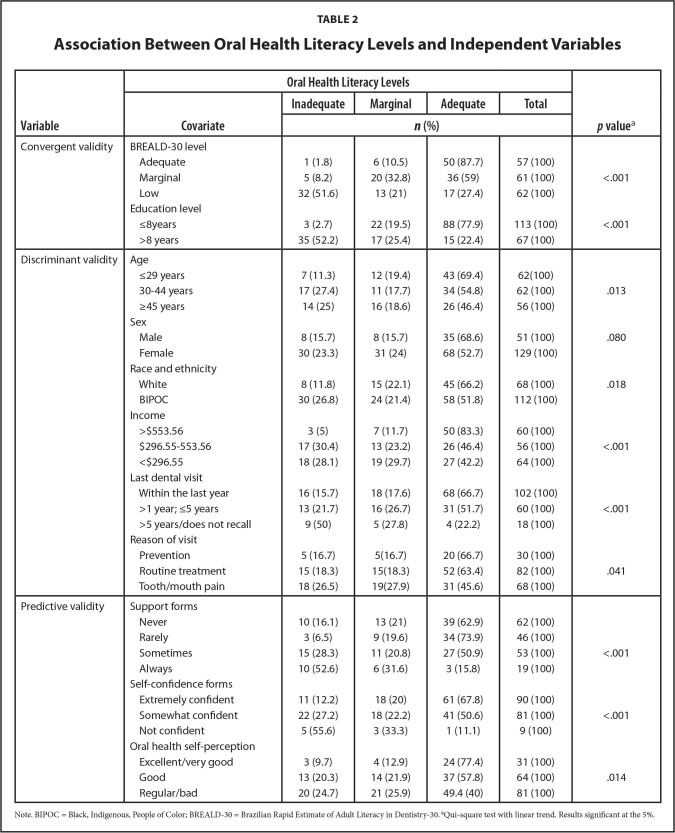
Association Between Oral Health Literacy Levels and Independent Variables

**Variable**	**Oral Health Literacy Levels**					***p* value** ^a^

	**Covariate**	**Inadequate**	**Marginal**	**Adequate**	**Total**	

		***n* (%)**				

Convergent validity	BREALD-30 level					
	Adequate	1 (1.8)	6 (10.5)	50 (87.7)	57 (100)	
	Marginal	5 (8.2)	20 (32.8)	36 (59)	61 (100)	<.001
	Low	32 (51.6)	13 (21)	17 (27.4)	62 (100)	
	Education level					
	≤8years	3 (2.7)	22 (19.5)	88 (77.9)	113 (100)	<.001
	>8 years	35 (52.2)	17 (25.4)	15 (22.4)	67 (100)	

Discriminant validity	Age					
	≤29 years	7 (11.3)	12 (19.4)	43 (69.4)	62(100)	
	30–44 years	17 (27.4)	11 (17.7)	34 (54.8)	62 (100)	.013
	≥45 years	14 (25)	16 (18.6)	26 (46.4)	56 (100)	
	Sex					
	Male	8 (15.7)	8 (15.7)	35 (68.6)	51 (100)	.080
	Female	30 (23.3)	31 (24)	68 (52.7)	129 (100)	
	Race and ethnicity					
	White	8 (11.8)	15 (22.1)	45 (66.2)	68 (100)	.018
	BIPOC	30 (26.8)	24 (21.4)	58 (51.8)	112 (100)	
	Income					
	>$553.56	3 (5)	7 (11.7)	50 (83.3)	60 (100)	
	$296.55–553.56	17 (30.4)	13 (23.2)	26 (46.4)	56 (100)	<.001
	<$296.55	18 (28.1)	19 (29.7)	27 (42.2)	64 (100)	
	Last dental visit					
	Within the last year	16 (15.7)	18 (17.6)	68 (66.7)	102 (100)	
	>1 year; ≤5 years	13 (21.7)	16 (26.7)	31 (51.7)	60 (100)	<.001
	>5 years/does not recall	9 (50)	5 (27.8)	4 (22.2)	18 (100)	
	Reason of visit					
	Prevention	5 (16.7)	5(16.7)	20 (66.7)	30 (100)	
	Routine treatment	15 (18.3)	15(18.3)	52 (63.4)	82 (100)	.041
	Tooth/mouth pain	18 (26.5)	19(27.9)	31 (45.6)	68 (100)	

Predictive validity	Support forms					
	Never	10 (16.1)	13 (21)	39 (62.9)	62 (100)	
	Rarely	3 (6.5)	9 (19.6)	34 (73.9)	46 (100)	
	Sometimes	15 (28.3)	11 (20.8)	27 (50.9)	53 (100)	<.001
	Always	10 (52.6)	6 (31.6)	3 (15.8)	19 (100)	
	Self-confidence forms					
	Extremely confident	11 (12.2)	18 (20)	61 (67.8)	90 (100)	
	Somewhat confident	22 (27.2)	18 (22.2)	41 (50.6)	81 (100)	<.001
	Not confident	5 (55.6)	3 (33.3)	1 (11.1)	9 (100)	
	Oral health self-perception					
	Excellent/very good	3 (9.7)	4 (12.9)	24 (77.4)	31 (100)	
	Good	13 (20.3)	14 (21.9)	37 (57.8)	64 (100)	.014
	Regular/bad	20 (24.7)	21 (25.9)	49.4 (40)	81 (100)	

Note. BIPOC = Black, Indigenous, People of Color; BREALD-30 = Brazilian Rapid Estimate of Adult Literacy in Dentistry-30.

a Qui-square test with linear trend. Results significant at the 5%.

### Exploratory Factor Analysis

Preliminary tests showed that data were suitable for PCA (K*MO* = 0.75; Barlett's Test of Sphericity significant [*p* < .001]). There were six factors with Eigenvalues higher than one, which explained 57.1% of the total variance. This was an unfeasible number of factors considering the length of the instrument and its theoretical rationale. Initially, a single-factor solution was explored, which explained only 21% of the total variance and presented eight items with low factor loadings (<0.40). However, as the original OHL-AQ authors (Sistani et al., 2014) suggested the existence of four components (reading comprehension, numeracy, listening and decision-making), a four-factor solution was tested, which explained 44.6% of the total variance. The factor loadings of a single and a four-factor solution are shown in **Table 3**.

**Table 3:**
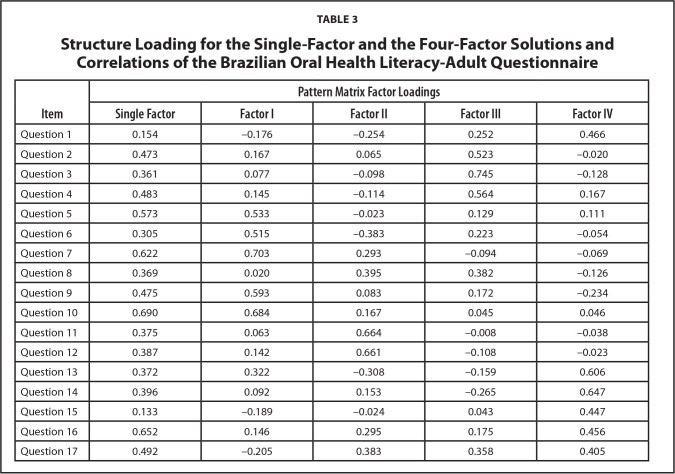
Structure Loading for the Single-Factor and the Four-Factor Solutions and Correlations of the Brazilian Oral Health Literacy-Adult Questionnaire

**Item**	**Pattern Matrix Factor Loadings**				
	**Single Factor**	**Factor I**	**Factor II**	**Factor III**	**Factor IV**
Question 1	0.154	−0.176	−0.254	0.252	0.466
Question 2	0.473	0.167	0.065	0.523	−0.020
Question 3	0.361	0.077	−0.098	0.745	−0.128
Question 4	0.483	0.145	−0.114	0.564	0.167
Question 5	0.573	0.533	−0.023	0.129	0.111
Question 6	0.305	0.515	−0.383	0.223	−0.054
Question 7	0.622	0.703	0.293	−0.094	−0.069
Question 8	0.369	0.020	0.395	0.382	−0.126
Question 9	0.475	0.593	0.083	0.172	−0.234
Question 10	0.690	0.684	0.167	0.045	0.046
Question 11	0.375	0.063	0.664	−0.008	−0.038
Question 12	0.387	0.142	0.661	−0.108	−0.023
Question 13	0.372	0.322	−0.308	−0.159	0.606
Question 14	0.396	0.092	0.153	−0.265	0.647
Question 15	0.133	−0.189	−0.024	0.043	0.447
Question 16	0.652	0.146	0.295	0.175	0.456
Question 17	0.492	−0.205	0.383	0.358	0.405

### Confirmatory Factor Analysis

**Figure 1.**
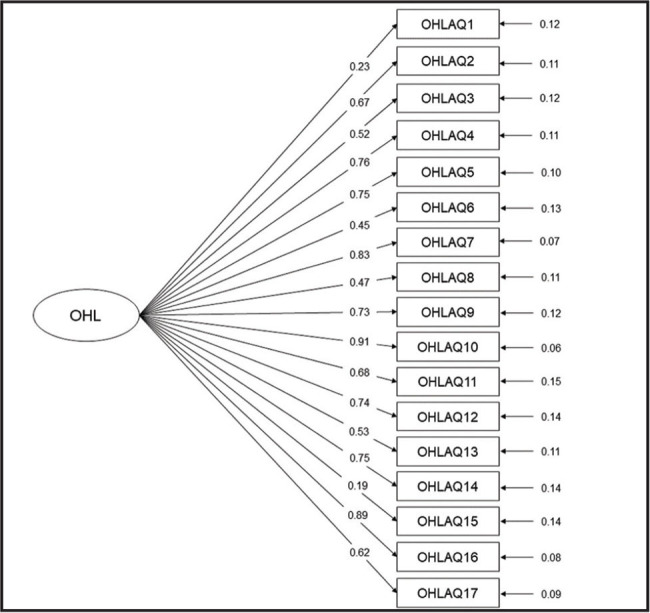
Confirmatory factor analysis of multidimensionality of the Brazilian Oral Health Literacy-Adults Questionnaire.

The four-factor solution of the BOHL-AQ was confirmed by a second-order CFA. The goodness of fit statistics was χ^2^ = 636.587, *p* < .001, C*FI* = 0.97, T*LI* = 0.97, and RMS*EA* = 0.03, indicating an excellent model fit. Factor loadings were >0.40 for almost all items, except for items 1 and 15 (**Figure 1**). Considering that two items of the instrument did not reach substantial loading (>0.40) on the proposed model, another model was tested without them. The goodness of fit statistics for the model with four factors without items #1 and #15 was X2 = 620.801, *p* < .001, C*FI* = 0.98, TLI =0.98, and RMS*EA* = 0.02, and the smallest factor loading was 0.435.

## Discussion

The cross-cultural adaptation and validation process of an instrument to be used in a new culture requires appropriate methodology, regarding the equivalence between the source and target language, the original content maintenance, the psychometric characteristics, and validity for the population for which it is intended (Praveen et al., 2021). To be used in the Brazilian context, we conducted the BOHL-AQ validation following rigorous methodological criteria. Initially, the study was authorized by the original OHL-AQ authors (Sistani et al., 2014), who applied the instrument outside the dental clinical settings, to avoid selection bias. They argued that most studies have been conducted at university dental clinics, where patients are better informed about oral diseases prevention and the risks of their decisions (Blizniuk et al., 2014; Holtzman et al., 2017; Mohammadi et al., 2018; Sistani et al., 2014). Thus, we planned the present study to be conducted in a sample of university employees and conducted the interviews inside the staff recreation rooms. The instrument completion time was faster (mean time = 8 minutes), comparing to Test of Functional Health Literacy in Dentistry (30 minutes) (Gong et al., 2007) and OHLI (20 minutes) (Sabbahi et al., 2009). The BOHL-AQ demonstrated satisfactory internal consistency (Cronbach's alpha = 0.73), similar to the original instrument = 0.72 (Sistani et al., 2014), its Hindi = 0.70 (Vyas et al., 2016), English = 0.74 (Flynn et al., 2016) and Mandarin version = 0.77 (Ho et al., 2019); excellent reproducibility (I*CC* = 0.97), whereas the ICC for the original instrument was 0.84 (Sistani et al., 2014) and the Hindi version was 0.94 (Vyas et al., 2016). Participants with high education level had also significantly high OHL level (convergent validity), similar to the original instrument and other studies that used OHL-AQ (Flynn et al., 2016; Ho et al., 2019; Mohammadi et al., 2018; Vyas et al., 2016). Individuals who self-declared more independent and confident on reading and filling out health forms, those with better self-perception on oral health (predictive validity), and those individuals who visited the dentist during the last year for preventive reason (discriminant validity) also showed high OHL levels, as observed in other studies using Brazilian OHL instruments (Cruvinel et al., 2017; Junkes et al., 2015). Dimensionality is a psychometric property not explored in the original OHL-AQ, but it is a fundamental property of instrument scores because it determines the number of required scores to adequately characterize the construct. Based on some EFA fit indices, the OHL-AQ English version authors considered that OHL instrument measured a single factor, while other results from the same study, as the identification of groups of highly correlated variables, did not support this conception (Flynn et al., 2019). Our findings suggest a four-dimensional structure, in line with four conceptual OHL components: reading comprehension, numeracy, listening and decision-making. Some authors have held that OHL comprises up to seven dimensions, including additional skills such as communicating with health care providers and the ability to navigate the health care systems (Dickson-Swift et al., 2014; Jones et al., 2014). Although consensus has not been established, most data indicate substantial correlation (i.e., that OHL could be adequately summarized with one total score) (Flynn et al., 2019). More studies are necessary to establish the predictive validity of the BOHL-AQ and to design and implement interventions that effectively manage the effect of OHL.

## Study Limitations

Although a randomly selected sample would be preferable, representativeness is not required for psychometric analysis. The findings could be generalized to the Brazilian adult population, but external validation on a larger sample should be done. Future studies are suggested to be conducted in other Brazilian regions, among underprivileged individuals and those with low OHL levels. Further analyses should also include a detailed item-by-item comparison of other OHL-AQ language versions to determine if values are impacted by cross-cultural differences.

The present study supports the theory of OHL multidimensionality and provides good reliability and validity findings. The BOHL-AQ proved to be a simple and reliable instrument that can be used for screening at clinical and community Brazilian settings to identify individuals with different degrees of comprehensive functional OHL.
